# Barriers and facilitators to the uptake of the Ischaemia with Non-Obstructive Coronary Arteries (INOCA) recommendation by cardiologists in the Netherlands: A qualitative study

**DOI:** 10.1016/j.ijcrp.2025.200480

**Published:** 2025-07-29

**Authors:** Linda Modderkolk, Irene Göttgens, Lori van den Hurk, Sabine Oertelt-Prigione

**Affiliations:** a Gender Unit, Department of Primary and Community Care, Radboud University Medical Centre, Nijmegen, Netherlands; b AG 10 Sex- and Gender-sensitive Medicine, Medical Faculty OWL, University of Bielefeld, Bielefeld, Germany

## Abstract

**Background:**

Ischemia with No Obstructive Arteries (INOCA) is a condition characterized by an elusive diagnosis and a significant impact on patients' quality of life. Recent evidence challenges previous assumptions about INOCA's benign prognosis, emphasizing the increased downstream risks associated with condition. A 2020 Dutch Society for Cardiology (NVVC) recommendation aims to guide cardiologists in the management of INOCA, but its adoption in practice varies. This study explores the interconnecting factors influencing the uptake of the INOCA recommendation.

**Methods:**

A qualitative interview study was performed to investigate cardiologists' adoption of the 2020 NVVC INOCA recommendation in the Netherlands, utilizing the Theoretical Domains Framework and COM-B model. A diverse sample of Dutch cardiologists was recruited and digital semi-structured interviews were conducted and analyzed using directed content analysis.

**Results:**

A total of 14 Dutch cardiologists (6 women, 8 men) was interviewed to explore factors influencing their uptake of the 2020 NVVC INOCA recommendation. The immediate influences on uptake were grouped into three domains: capabilities, opportunities, and motivation according to the COM-B model. Most importantly, underlying assumptions about the evidence base, diagnostic accuracy, and gendered stereotypes significantly influenced recommendation uptake.

**Conclusions:**

Underlying assumptions about the disease entity and the affected patient, which are seldom investigated in implementation research, significantly affect the overall uptake of the INOCA guideline. Careful investigation of these assumptions is necessary to challenge them and foster an environment conducive to the uptake of structural implementation measures.

## Highlights

• INOCA's prognosis is more severe than previously assumed, impacting health outcomes.
• Cardiologists' INOCA recommendation uptake varies, influenced by multiple factors.
• Key barriers include evidence skepticism, diagnostic doubts, and gender biases.
• Addressing underlying assumptions is crucial for effective recommendation implementation.

## 1 Introduction

Ischemia with no obstructive arteries (INOCA) is a clinical entity that has received increasing attention in recent years. Patients with INOCA show functional evidence of ischemia frequently without detectable obstructive artery disease (CAD) upon classic angiography. Patients often experience a considerable decline in quality of life without having obtained a clear clinical diagnosis and specific treatment by their cardiologists [[1], [2], [3]]. While it is difficult to define the overall prevalence of INOCA, it is estimated that 3 to 4 million people in the United States suffer from angina pectoris without obstructive coronary disease [4], with a significant predominance of women [[4], [5], [6], [7]]. The current prevalence of the disease in the Netherlands remains undetermined [8]. The Netherlands Registry for Invasive Coronary Vasomotor Function Testing (NL-CFT), launched in 2021, has so far enrolled over 1,200 patients across 15 secondary and tertiary centers. Vasomotor dysfunction occurs in approximately 78 % of the referred patients with angina, demonstrating how the procedures are both safe and diagnostically robust in routine clinical practice [9,10]. Notably, findings from a comprehensive Swedish registry indicate that nearly 80 % of women under the age of 60 presenting with stable angina symptoms exhibited no discernible coronary obstructions upon angiography, compared to 40 % of men with the same symptoms [11].

Historically, INOCA was assumed to bear a benign prognosis. However, recent evidence highlighted an increased risk of adverse cardiac events, recurrent cardiac symptoms, and associated hospitalizations, as well as a 10-fold increase in heart failure with preserved ejection fraction (HFpEF), stroke, and coronary microvascular dysfunction (CMD) in these patients [6,12,13]. Following these insights, a practice recommendation to support cardiologists in recognizing, diagnosing, and treating patients with suspected INOCA was developed in 2020 by the Working Group on Gender within the Dutch Society for Cardiology (NVVC) [14,15]. Nevertheless, implementation of clinical guidelines and recommendations in the Netherlands remains unpredictable, slow, and complex, with a limited use of structured implementation plans and evaluation procedures leading to inconsistent guideline use and notable differences between hospitals [16,17].

Given the negative impact of INOCA on patients' health and quality of life, gaining insight into cardiologist behaviour regarding the recognition, diagnosis, and management of this condition within the Dutch healthcare context is crucial. The Netherlands features a dual hospital system (academic and peripheral), a strong emphasis on cost-effective and evidence-based care, and central guideline committees, yet guideline adherence can vary. No data exist on how Dutch cardiologists interpret and implement INOCA recommendations, nor about barriers specific to the national professional and institutional setting. Therefore, this study aims at identifying the factors that influence the uptake of the novel INOCA recommendation by cardiologists in the Netherlands and at providing suggestions for interventions to support its diffusion and implementation.

## 2 Methods

### 2.1 Study design

A qualitative interview study was performed to identify behavioral barriers and facilitators impacting cardiologists' uptake of the 2020 NVVC INOCA recommendation in The Netherlands. The study's design was informed by Atkin's TDF guide and Michie's COM-B Model [18,19].

### 2.2 Participants and sample size

We recruited cardiologists working within a Dutch hospital or outpatient clinic. Inclusion criteria were: active practice as a cardiologist within a Dutch hospital or clinic, fluency in Dutch, and provision of informed consent. Lack of compliance with any of these criteria led to exclusion from the study. Following the principles for operationalization of data saturation in theory-based studies by Francis et al., the initial analysis sample was set at a minimal sample size of 10 cardiologists [20]. To ensure data saturation we set a stop criterion at four participants, i.e. once four additional interviews beyond the minimal sample size provided no substantial new data, recruitment was terminated. This approach is considered appropriate for qualitative, theory-driven research aiming to explore perspectives in depth rather than develop generalizations to larger populations. A convenience sample was recruited including cardiologists who already employ the recommendation in their practice and cardiologists who do not, to enhance content validity. To further increase diversity, we aimed for variation in age, gender identity, and affinity with the INOCA topic. While diversity in region or practice type was not systematically ensured, this was partially achieved through the recruitment strategy. Cardiologists were invited to participate in the study by means of a newsletter of the Dutch Society of Cardiology (NVCC). We also invited cardiologists using a snowball approach through members of the ‘Gender workgroup’ of the NVVC or cardiologists who participated in the study themselves. Our sample was intentionally limited to cardiologists practicing in the Netherlands to ensure contextual relevance to the Dutch healthcare system.

### 2.3 Reporting patient and public involvement

Patients were not directly involved in this study; however, its initiative was informed by the experiences of patients with INOCA.

### 2.4 Theoretical framework

#### 2.4.1 COM-B model and the Theoretical Domains Framework

**Fig. 1:**
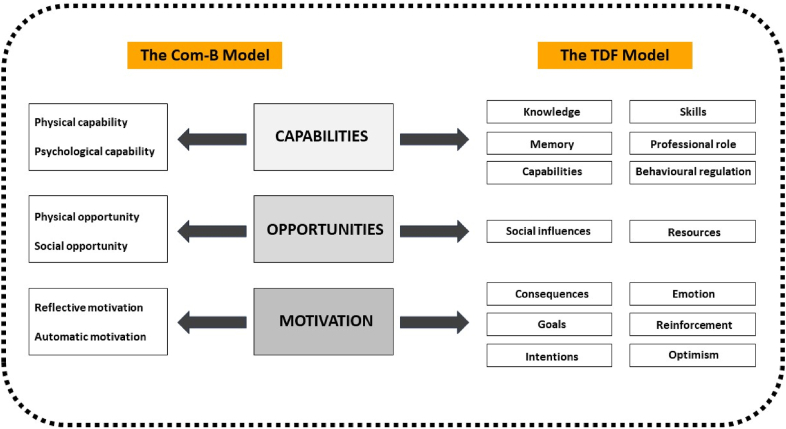
The combined COM-B/TDF model.

We designed this study using the Theoretical Domains Framework (TDF), which encompasses 14 theoretically based domains impacting behavioural change (See Fig. 1.). [21] The TDF can identify structural and psychological factors impacting healthcare providers' behaviour, especially during the implementation of new practices. The COM-B model is a simplified version encompassing three essential components: Capabilities, Opportunities, and Motivation (COM) (See Fig. 1.). These three components interact with each other and are crucial to any intervention that requires behavioural change (-B). Capabilities refer to a person's ability to engage in the desired behaviours and can be divided into physical (e.g. knowledge) and psychological (e.g. decision-making abilities). Opportunities refer to all external circumstances, such as physical (e.g. resources like time) and social (e.g. the influence of peers), that determine the possibility of performing the desired behaviour. Motivation comprises reflective and conscious drivers (e.g. goal setting), as well as emotional responses (e.g. frustration or optimism.) [19,22] For this study, we mapped the 14 TDF domains onto the COM-B model, as demonstrated by Atkins (2016), to allow for rigorous analysis of the collected data while maintaining focus during the interview process. We will further refer to the combined framework in the reporting as COM-B/TDF [22].

### 2.5 Data collection

One researcher in the team (LM) conducted all digital semi-structured interviews between December 2022 and March 2023. The interviews lasted 20–30 min, they were audio recorded and transcribed verbatim guaranteeing anonymization of all personal data. In line with the COM-B structure, the interviews focused on the target behavior: “*Do cardiologists consult and follow the NCCV INOCA recommendation for identification, diagnosis and treatment of patients with suspected coronary vascular dysfunction?*”. The interview guide was developed in collaboration with two cardiologists and a policy advisor to ensure clinical and policy relevance. Although no formal pilot test was conducted, we adjusted the guide after the first four interviews to optimize the questions’ specificity (Supplement 1). We used follow-up probes and questions to deepen our insights into the domains relevant to the target behaviour.

### 2.6 Data analysis

We employed directed content analysis to identify domains of the COM-B/TDF relevant to the specified target behaviours [23]. Hence, we employed a deductive approach, where the coding framework was derived from the COM-B/TDF model. The researchers created definitions for each domain to determine how they apply to the INOCA recommendation (see Supplement 2). Subsequently, all data was read and coded independently by two researchers (LM, LvdH) using Atlas.ti 9 software and discussed until consensus was achieved. We used the Consolidated Criteria for Reporting Qualitative Studies (COREQ) checklist for data reporting [24].

#### 2.6.1 Research team and reflexivity

LM is a female Ph.D. researcher with an extensive experience in qualitative research. IG is a female post-doc researcher in gender-sensitive medicine with experience in (pre-)implementation studies. LvdH is a female research assistant with qualitative research experience. SOP is a female professor of sex- and gender-sensitive medicine with an internal medicine and public health background.

## 3 Results

A total of 14 cardiologists (6 women and 8 men) were interviewed. Most participants were employed in peripheral hospital settings and were fully or partially familiar with the INOCA recommendation (Table 1).

**Table 1:** Selected participants characteristics.

	Overall (N = 14)	Women (N = 6)	Men (N = 8)
**Age**			
30–39 years	3	2	1
40–49 years	3	2	1
50–59 years	6	1	5
60+ years	2	1	1
**Profession**			
Cardiologist	12	6	6
Interventional cardiologist	2	0	2
**Work setting**			
Peripheral hospital	12	5	7
Academic hospital	1	1	0
Clinic	1	0	1
**Uptake level of the INOCA recommendation**			
Fully familiar and following recommendation	5	2	3
Partially familiar and following recommendation	5	3	2
Deviating from the recommendation	3	1	2
Not familiar with recommendation	1	0	1

### 3.1 Barriers and facilitators impacting INOCA recommendation uptake

Overall, the defined target behaviour was not “achieved” or “not achieved”. Instead the reported implementation practices have to be described as a spectrum. “Following the recommendation” spanned from (a) knowing its content and following the recommended diagnostic and treatment road map, to (b) partially knowing and following it, to (c) deviating from it (knowingly or unknowingly) when deemed necessary, to (d) not being familiar with or following it. When focusing on the COM-B/TDF model — capabilities, opportunities, and motivation—the positioning of cardiologists along the spectrum is due to various barriers and facilitators. It is essential to note that barriers and facilitators towards the adoption of the recommendation do not inherently correlate with the willingness to embrace the recommendation. Cardiologists willing to follow the recommendation or implementing it could still experience barriers in doing so. Overall, 19 facilitators and 26 barriers were mentioned in the interviews (see Supplement 3 for the overview). Illustrative quotes are presented in Table 2.

**Table 2:** Exemplar quotes of factors impacting recommendation uptake.

COM-B components	Facilitators impacting recommendation uptake	Barriers impacting recommendation uptake
**Capabilities (physical and psychological)**	*‘You always start by ruling out obstructive coronary disease. I didn't do that at first. First, I was just going to do a perfusion scan. […] A good test didn't rule it out and now you actually very often skip that step of first doing a perfusion scan for example or the cycling test. You just start with obstructive coronary disease or not. Either with a catheterization or a CT and you start working from there, actually. And if someone does have obstructive coronary artery disease, you start treating that first and then see what remains of the symptoms. […] So, you think it's actually the reverse of what I did first. And this is really much more logical, and it also just gives me a lot of guidance in practice to deal with it.’* (R1) I: *‘What makes the recommendation easy for you to find?’ R*: *‘Because I have it on my desktop somewhere. I know exactly where it is.’* (R6) I: *‘So how do you do that when you get INOCA patients in your outpatient clinic? Sometimes you know in advance, sometimes you may not. How do you organize that?’ R: 'What I usually do then […] is that you just then put it at the end of your consultation hour. Usually I don't put it in the middle of my consultation hour with a double spot, because I don't like that very much because there is already a lot of pressure anyway and then it's better to just take it at the end of your consultation hour and then just say: well, I'll schedule you at the end of the consultation hour, but then I'll just have the time to consult with you quietly and I won't feel the pressure from the staff that there is already another patient waiting or something like that.’* (R12)	*‘More focused treatment I'm still missing. So that you say well, you have this form. And then that will work. And if they still have complaints that I can really say, well, that doesn't matter. You have to learn to live with that. That bit of uncertainty that you still have in the treatment, you pass on to the patients. I hope that in five years’ time we will be able to provide a little more clarity about this. Because people really like knowing where they stand.’* (R6) *‘Well, looking at it this way, I think the pocket chart is very full of information. And there's also a lot of information in there that, I think, is not directly super relevant in people with chest pain without obstructive coronary disease and that does make it a little bit difficult. So, I think that, reading it like that, I feel like there's a lot of information put into that pocket chart which does reduce the clarity a little bit.’* (R3) *‘In terms of skills, I have mastered it so far, I know what the recommendation means and I understand what needs to be done. It remains elusive. That's what I said at the beginning. Because these are very diverse symptoms. And that also makes this very difficult. And that's also partly why maybe people stay away from it. It's not a typical story. Look, if you break your arm, you have a broken bone. That's obvious. If you have a heart attack, you have a clogged blood vessel. That's also obvious. And this is much more elusive. And the symptoms are very diverse. And that makes it more difficult. And I haven't quite mastered that either. Nobody does. You will always miss things and you will always treat people who don't have it as well. That will continue to be difficult.’* (R7)
**Opportunities (physical and social)**	*‘Because at the moment our professor is very much involved in it, so you do see that it affects the entire department. We also have it as one of our focus points, especially INOCA in cardiovascular suffering, so you see more and more also the older cardiologists opening up to it.’* (R2) I: *‘Are there external factors that influence whether you follow this guideline? Is there something to do with time or with money, with resources, with opportunities?’* R: *‘Yes, the tests are readily available with us, of course. That makes a difference.’* (R7) *‘And I have expertise within my department, so I must say that I also do consult quickly, say, by phone or through action lines whether I refer people.’* (R2)	*‘Because it's quite a difficult patient group that also involves a lot of emotion. What you do also involves a lot of psychology. If that is not your cup of tea as a doctor, then I can also imagine that you are less likely to go down this path with such a patient.’* (R1) *‘Those people have been walking around for years with very intense symptoms, they just don't know when to reach out. They don't dare to call the ambulance, because they know the ambulance will come and say: here she is again.’* (R10) *‘I can certainly imagine at the time of starting a women's consultation, I think the word is a bit unfortunate. I think because it's also men. That's an emancipation problem which is a bit to the detriment of men I think.’* (R5) I: *‘What might be barriers for you?’* R: *‘To use it?’* I: *‘Yes.’* I: *‘Yes. That's a good question. There are quite a few. Look, I think the busyness of your consulting hours is a barrier to start using it right away.’* (R3)
**Motivation (reflective and automatic)**	*‘You don't ignore people. You can help patients more, offer more than without the recommendation. […] You can offer them the current state of the science and knowledge and try to figure out as best as you can what they have. […] Instead of it being a dead end of hey here it stops, we can move forward.’* (R7) *‘I really enjoy doing outpatient work. So, I can always get a lot of satisfaction from improving patients′ [issues] or at least being open to patients. So, I just really enjoy focusing on it.’* (R12) *‘It is frustrating when people have complaints but don't get the help they need. As a medical professional, I sometimes blame myself for patients I sent home who may have had an underlying condition that I didn't diagnose. These conditions may not be immediately life-threatening, but they can still cause long-term problems. Although this is inherently part of the work, if you can do a little more in that and diagnose people, that's very nice.'* (R10)	*‘I also notice, fortunately, that people are becoming more critical. I mean I'm quite willing, because I'm an interventional cardiologist, to do examinations as well. I'm quite willing to use my hands and also do catheterizations, I don't mind that kind of thing either, but it always remains a risk for patients. I think if it's not necessary, you should avoid it.’* (R9) *‘But it's not an easy category. It's not like, well, you know I see it as INOCA, I give three tablets and then it's done. Surely the practice is a little more complex. I notice that this does not always lead to the complete disappearance of the problem. And I still find that a bit tricky sometimes.’* (R12) *‘I have that consultation hour* [INOCA consultations] *on Friday mornings and I find it tiring sometimes. They* [the patients] *really do take energy.’* (R6) *‘I mean 80 % is psychology what's going on. Not just psychology, but context. Technique is very important, but is only a very small piece. And the recommendation focuses very much on that technique. […] At least women come out of a kind of desperation of who have had symptoms for years, ‘but if I get an adenosine test … ’ Then I say but it's a diagnostic. Then you haven't been treated. Whereas very often it is brought- Again, I think that's another disadvantage of the recommendation in general, as a kind of holy grail of if you just follow that then the problem is solved. Then you may have only identified the problem. And then it starts.’* (R5)

I = Interviewer.

R = Respondent.

#### 3.1.1 Capabilities (physical and psychological)

##### 3.1.1.1 Facilitators

Within the capabilities component of COM-B, knowledge appears as the most prominent domain towards the uptake of the recommendation. A majority of cardiologists state that the recommendation provides the necessary scientific knowledge for a clear diagnostic and treatment process. This increases their understanding of the condition and helps structure their work. Taking patients with INOCA seriously and revisiting the recommendation as often as necessary to ensure optimal treatment are seen as professional responsibilities. Participants feel that they have the necessary skills to execute the required steps in the recommendation and the interpersonal skills for effective communication with their patients. Some cardiologists demonstrate a proactive approach, such as keeping the recommendation always accessible and distributing the care for patients with INOCA among the team members.

##### 3.1.1.2 Barriers

On the other end of the spectrum, several cardiologists expressed that current scientific knowledge for diagnosis and treatment is insufficient and, hence, decreases the uptake of the recommendation. The supposed lack of a scientific basis impacts their perceived ability to detect symptoms and make clinically sound decisions. For some others, however, the amount of information presented in the recommendation impairs its efficient use. One cardiologist was completely unfamiliar with the recommendation. Some participants pointed out that a recommendation carries less weight than a guideline, thereby decreasing their willingness to follow it. Several cardiologists regard it as their professional role to diverge from the recommendation, when necessary, based on their clinical judgment. Some cardiologists also prefer to refer patients with suspected INOCA to colleagues with more expertise or interest in the topic. This approach was often connected to a perceived lack of necessary interpersonal communication skills for the interaction with patients with INOCA in the midst of diagnostic and treatment uncertainty.

#### 3.1.2 Opportunities (physical and social)

##### 3.1.2.1 Facilitators

The role of colleagues and INOCA experts recommending the uptake of INOCA care was essential in domain of social influences. Division of INOCA care within a team has a positive impact on uptake, especially when colleagues in a hierarchically superior position emphasize its importance. Interacting with knowledgeable experts also has a positive impact on uptake because it enhances the perceived relevance of the condition.

##### 3.1.2.2 Barriers

The patient population is described by most participants as a complex, demanding, and well-informed group requiring time and attention for a condition that affects them physically and psychologically. This is perceived as demanding and at times tiring by cardiologists.

All cardiologists describe the patient group as predominantly consisting of women and stress the importance of specific sex differences in the onset and treatment of symptoms. Several cardiologists remarked that there is also a relevant group of men with suspected or diagnosed INOCA. In some cases, the general image of a patient with INOCA is described as ‘a typical woman', i.e. an often highly educated woman with anginal symptoms and normal coronary arteries reporting significant psychological symptoms. A few cardiologists reported that the complex care needs of this specific population can hinder their motivation to engage with these patients, and reduce the relevance they attribute to the condition. Furthermore, a limited number of cardiologists feared that the male predominance in cardiology may reduce attention to INOCA, as it is considered a women's issue.

A few cardiologists have raised the concern that men with INOCA may face social stigma given that the overall image of the condition is primarily centered around women. Some cardiologists have expressed their reluctance towards INOCA because they feel that the topic is being promoted by women cardiologists who are advocating for its recognition. Additionally, a small number of cardiologists have pointed out that the recommendation lacks attention to the influence of the personal life circumstances of patients on their condition, such as their job or care tasks, focusing mostly on the technical care aspects and falling short of a holistic approach.

#### 3.1.3 Motivations (reflective and automatic)

##### 3.1.3.1 Facilitators

A majority of cardiologists felt that the recommendation provides clarity about the diagnostic and treatment process for INOCA, increasing its uptake and the acknowledgment of the condition among colleagues. Better recognition of patients' symptoms and the ability to propose a step-by-step diagnostic process is perceived as supportive in increasing patients′ acceptance of their condition. Some cardiologists explicitly describe helping patients accept the impact of their diagnosis as a professional goal, which can be achieved by applying the recommendation. In general, for those working in clinical practice, uptake of the recommendation is strongly motivated by the expectation to improve the quality of patient care.

##### 3.1.3.2 Barriers

Two main concerns that prevent recommendation uptake are connected to its expected outcomes: a fear of unnecessary testing and overdiagnosis, and a lack of sufficient knowledge about the needs and availability of follow-up care after INOCA diagnosis. Several cardiologists express concern about the amount of invasive diagnostic testing for INOCA and the reliability of the outcomes given the early stage of the diagnostics.

The other concern relates to the follow-up care for patients after INOCA diagnosis. Several cardiologists stress the importance of multidisciplinary follow-up care addressing all dimensions of life, as the condition highly impacts the quality of life. Having insufficient resources and limited treatment options to support patients is challenging and requires much time and energy during consultations. Some describe it as a frustrating experience for both patients and cardiologists, which limits the motivation among some cardiologists for (further) uptake of the recommendation.

#### 3.1.4 Compounding factors impacting the uptake of the INOCA recommendation

**Fig. 2:**
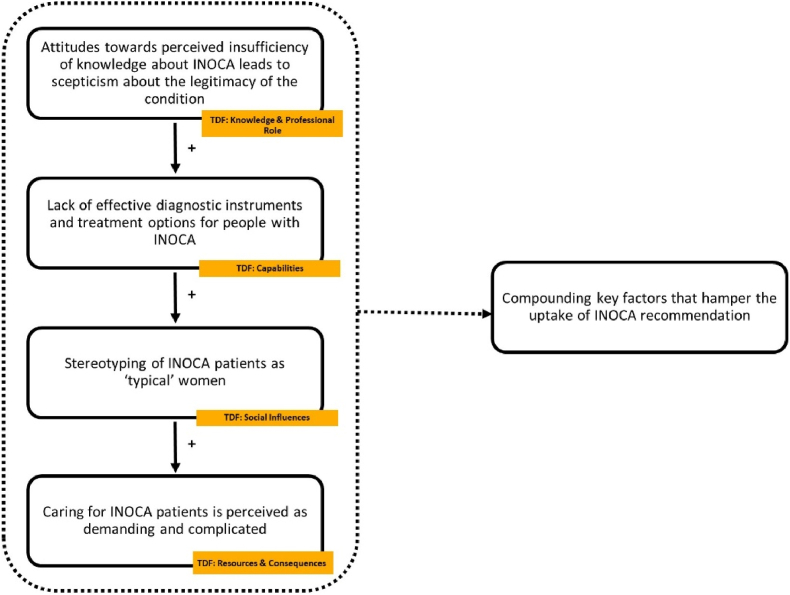
Compounding key factors that hamper the uptake of INOCA recommendation.

**Fig. 3:**
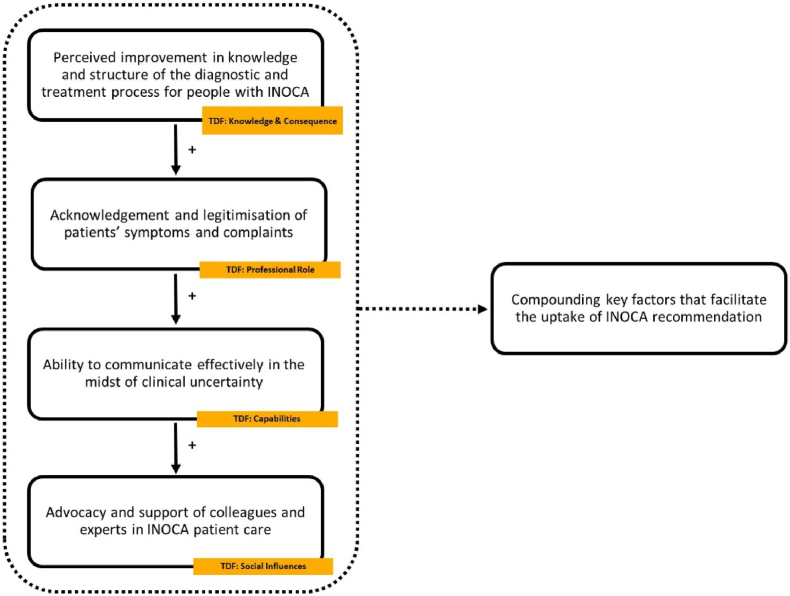
Compounding key factors that facilitate the uptake of INOCA recommendation.

Analysis of the data showed that the various factors are not only relevant in isolation but can reinforce each other through their interaction. Furthermore, some key factors that determine the uptake seem to be the underlying assumptions about INOCA itself that drive attitudes and behaviours (Fig. 2, Fig. 3).

Fig. 2 describes the interaction of four different factors that belong to 6 TDF domains. When the perception of insufficient knowledge, diagnostic tools, and treatment options, is combined with a stereotyped image of patients as ‘typical’ women who require demanding care, the willingness of cardiologists to engage with this group decreases.

Fig. 3 depicts compounding factors in 5 TDF domains that facilitate recommendation uptake. The perceived increase in diagnostic and treatment clarity, combined with the acknowledgment of the condition, helps to communicate effectively with patients, despite the remaining uncertainty related to the condition. This is mediated by the support perceived from colleagues and experts.

## 4 Discussion

The investigation of the uptake of the INOCA recommendation by cardiologists in the Netherlands revealed a complex interaction of facilitators and barriers leading to a spectrum of diverse implementation practices. Traditional implementation drivers in the domains of knowledge, motivation and structural opportunities, appeared to be significantly affected by pre-existing assumptions about INOCA itself and the patients experiencing it.

Cardiologists' opinions on structural factors influencing the uptake of the INOCA recommendation diverged significantly. Compared to other medical conditions [25], uncharacteristically strong opposing views characterized the perceptions of INOCA. Our interviews reflected this divide with some cardiologists reporting increased knowledge and clarity due to the recommendation, while others questioned its applicability and legitimacy compared to formal guidelines. Social and peer influences, such as colleagues, could either facilitate or hinder implementation. Stereotypes about patients with INOCA and cardiologists advocating for the topic were expressed, as well as challenges in long term care. Some participants also reported the risks of overdiagnosis and insufficient resources for follow-up. These divergent views seem driven by deeper assumptions about INOCA, with barriers and facilitators compounding each other's impact on uptake.

### 4.1 Role of assumptions in implementation science

‘*Assumptions*’ play a critical role for the successful implementation of evidence-based practices (EBPs), yet they remain challenging to define and measure consistently [26]. While the Theoretical Domains Framework (TDF) does not include a formal definition for this construct, in evidence-based medicine (EBM) ‘*assumptions*’ are generally understood as “*taken-for-*granted *beliefs or presuppositions that are often implicit and guide our clinical reasoning, construction of knowledge and the decision-making process in clinical situations*” [18,27]. It is, therefore, imperative to analyze these underlying assumptions to fully understand barriers and facilitators towards the uptake of the INOCA recommendation. Rather than discussing the stand-alone behavioral TDF domains that impact the uptake of the recommendation, we will describe three key underlying assumptions that impact multiple TDF domains, as enablers or inhibitors for the INOCA recommendation uptake.

The first underlying assumption relates to the clinical entity of INOCA itself. While the term INOCA is increasingly recognized, historically the condition was known as ‘Cardiac Syndrome X (CSX)’ and associated with a perceived benign prognosis [28]. INOCA remains a heterogeneous condition with persistent gaps in understanding of its pathophysiology, lack of cost-effective diagnostics, and variable prognoses based on underlying coronary microvascular dysfunction [29]. Some cardiologists reported growing knowledge as a driver, while others saw the lack of comprehensive evidence as a barrier, demonstrating opposing perceptions of the same phenomenon. Recent studies challenge the benign perception of INOCA, emphasizing its complexity [30]. Education is essential to close these knowledge gaps, and both single and multifaceted interventions show positive effects [31]. Cardiologists also highlighted the need to constantly update recommendations to reflect the latest evidence on INOCA given the dynamic developments in the field. “Living Guidelines,” where updates occur as new evidence emerges, could fulfill this request [[32], [33], [34]] and, alongside targeted educational efforts, productively engage skeptics.

The second underlying assumption is connected to the complexity of diagnosing INOCA. Diagnosis often requires invasive tests to confirm myocardial ischemia [28], particularly in cases of CMD, leading to concerns about both underdiagnosis and overdiagnosis. Diagnostic uncertainty also complicates treatment decisions, given the lack of overall consensus on treatment protocols [3]. This dual challenge—improving diagnostic accuracy and developing tailored treatment strategies—underscores the importance of recommendations like the NVVC INOCA recommendation and the EAPCI Expert Consensus Document [7]. Nevertheless, some cardiologists in our study cited the limited legitimacy of these recommendations compared to more rigorously developed guidelines as a reason for low uptake. Uncertainty in diagnosis and treatment often led them to refer patients to more experienced colleagues, avoiding deeper engagement with the recommendation.

The third underlying assumption highlights the potentially sex-specific and gendered nature of INOCA. INOCA is often described as a ‘women's disease’ due to the higher incidence of microvascular dysfunction among female patients. The patients are also described as ‘complex and demanding’, reiterating many gendered stereotypes that affect conditions preponderant in women [35]. In addition to being biased, this view can also marginalize male patients and oversimplify the disease's complexity [36]. While advocacy for women in cardiology is important given the field's male-dominated history, it should not overshadow the need for nuanced care across all gender identities.

A gender-sensitive approach to cardiology must recognize that ‘sex’ and ‘gender’ are distinct but interconnected constructs. Biological aspects like genetics and hormones interact with gender norms and roles, influencing cardiovascular outcomes [37]. Recent studies suggest that gender-related factors can counteract the effects of biological sex on clinical outcomes, highlighting the risks and disadvantages for women patients in a male-dominated cardiology field [38].

### 4.2 Strengths and limitations

A key strength of this study is the use of a theory-informed, qualitative approach to explore barriers and facilitators to the uptake of the INOCA recommendation among cardiologists. The study design allowed for an in-depth understanding of behavioural determinants relevant to clinical practice. In addition, this study explored and identified underlying assumptions about INOCA that influence professional behaviour. These assumptions are often overlooked but are essential to address in implementation efforts. Efforts were also made to ensure diversity in participants' age, gender identity, and affinity with the topic.

Several limitations should be acknowledged. Given the use of convenience and snowball sampling, both self-selection and selection bias cannot be ruled out. Cardiologists with a particular interest in INOCA may have been more likely to participate, and although we aimed for variation in participant characteristics, the sample may not fully represent the broader cardiology population. Furthermore, as the study focused on Dutch cardiologists, findings may not be directly transferable to other healthcare settings or cultural contexts. Finally, formal member-checking was not performed, which may have limited the opportunity to verify the interpretation of participants' views.

## 5 Conclusion

Our study is the first to identify underlying behavioral assumptions influencing adherence to the NVVC INOCA recommendation. Ignoring these assumptions will hinder the development of targeted interventions to improve uptake. Recruiting cardiologists with minimal affinity for INOCA was challenging, but we included a diverse range of participants to capture a broad perspective. While we achieved data saturation, the factors identified by the participating cardiologists may not be exhaustive. As the INOCA field evolves, new insights will likely emerge. Future research could build on our findings by using a quantitative approach, such as a national survey among cardiologists, to systematically examine diagnostic practices, treatment preferences, and the extent to which the identified perceptions and assumptions translate into clinical decision-making.

The knowledge gaps and assumptions around INOCA significantly influence how Dutch cardiologists engage with the INOCA recommendation. Recognizing INOCA's complexity can lead to more personalized care, but medical education and continuous professional development are crucial to keeping cardiologists updated on the latest INOCA research and management, enabling more acceptance and effective application of the recommendations.

Going forward, a multi-faceted approach is needed to enhance INOCA care. This includes advancing research, improving diagnostic tools, and developing comprehensive treatment strategies. Additionally, deconstructing current assumptions, especially those related to gender, is essential. A deeper, stereotype-free understanding of INOCA will support more equitable and effective care for all patients.

## CRediT authorship contribution statement

**Linda Modderkolk:** Writing – review & editing, Writing – original draft, Visualization, Software, Resources, Project administration, Methodology, Investigation, Formal analysis, Data curation, Conceptualization. **Irene Göttgens:** Writing – review & editing, Writing – original draft, Visualization, Formal analysis. **Lori van den Hurk:** Writing – review & editing, Formal analysis. **Sabine Oertelt-Prigione:** Writing – review & editing, Supervision, Resources, Methodology, Funding acquisition, Conceptualization.

## Availability of data and material

The datasets used and analyzed during the current study are available from the corresponding author upon reasonable request.

## Ethics approval and consent to participate

The study was approved by the institutional review board (IRB) of the Radboud University Medical Center (Dossier number: 2022–15850).

## Consent for publication

Not applicable.

## Declaration of competing interest

The authors declare no competing interests.
