# Supplementary material for: Barriers and facilitators to the uptake of the Ischaemia with Non-Obstructive Coronary Arteries (INOCA) recommendation by cardiologists in the Netherlands: A qualitative study
Source: Int J Cardiol Cardiovasc Risk Prev. 2025 Jul 29;27:200480. doi: 10.1016/j.ijcrp.2025.200480 (PMC12344192; doi:10.1016/j.ijcrp.2025.200480)
Supplement: Multimedia component 1 [file mmc1.docx]

**Supplement 1: The interview guide**

| **Topics** | **Probes** |
| --- | --- |
| 1. **PSYCHOLOGICAL CAPABILITY:**   **How familiar are you with the INOCA recommendation?**   - 1. **What do you know about it?**   2. **Do you use it in daily practice?**  1. **PHYSICAL CAPABILITY:**   **What is required or necessary to use the recommendation in your daily practice?** | - What do you know about the recommendations’ content? - Do you know how to implement the road map in daily practice? - What is necessary on a practical level to implement the recommendation? How easy/difficult is it to make that happen? - What changes in your working behavior when you implement the recommendation? What do you need to do (differently)? - How capable do you feel in executing the recommendation? |
| 1. **PHYSICAL OPPORTUNITIES:**   **Which resources (or the lack thereof) influence your choice to work according to the recommendation?**     1. **SOCIAL OPPORTUNITIES:**   **Which people in your working environment influence your choice to (not) implement the recommendation?** | - Resources: time, money, ICT facilities, location, access to diagnostic tests and treatment resources      - People: colleagues, other healthcare providers, work/department culture, management, patients, personal social circle |
| 1. **MOTIVATION:**   **What do you think the consequences will be if you follow the recommendation?**    **Which feelings play a role for you in implementing the recommendation?**    **What are your motivations for (not) implementing the recommendation?** | - What are the consequences for patients, yourself, professional group, others? - What are consequences in the short and long term? - Is there sufficient evidence? If not, what’s missing? - What are the costs of implementing the recommendation and do they balance out the benefits? - How prepared are you yourself to implement and to what extent do you feel pressured to implement the recommendation? - Are there things that interfere with or encourage implementation? - What is the culture regarding INOCA in your work environment? |
| **Is there anything else in relation to the implementation of the INOCA recommendation that I haven't asked about but that you think is important to mention?** |  |
