# Supplementary material for: Barriers and facilitators to the uptake of the Ischaemia with Non-Obstructive Coronary Arteries (INOCA) recommendation by cardiologists in the Netherlands: A qualitative study
Source: Int J Cardiol Cardiovasc Risk Prev. 2025 Jul 29;27:200480. doi: 10.1016/j.ijcrp.2025.200480 (PMC12344192; doi:10.1016/j.ijcrp.2025.200480)
Supplement: Multimedia component 2 [file mmc2.docx]

**Supplement 2: Definitions of the TDF domains related to the target behaviour**

| **COM-B components** | **TDF domain** | **Domain definition related to the INOCA recommendation** |
| --- | --- | --- |
| **Capabilities** | 1. Knowledge | Being up-to-date about the information in the recommendation about INOCA-related symptoms, the diagnostic process and recommended treatment options. |
|  | 1. Skills | Having the technical and interpersonal competences to execute the recommendation. |
|  | 1. Social/professional role and identity (Self-standards) | Deciding on the basis of implicit or explicit beliefs that the recommendation should guide their professional behavior. |
|  | 1. Beliefs about capabilities (Self-efficacy) | Being confident about their abilities to perform the necessary steps in the recommendation. |
|  | 1. Behavioral regulation | The personal and organizational change process necessary to follow the recommendation. |
|  | 1. Memory, attention and decision processes | The ability to meet the cognitive demands necessary to follow the recommendation. |
| **Opportunities** | 1. Environmental context and resources (Environmental constraints) | Material or immaterial assets impacting execution of the recommendation. |
|  | 1. Social influences (Norms) | People who influence(d) a cardiologist’s decision to follow the recommendation. |
| **Motivation** | 1. Beliefs about consequences (Anticipated outcomes/attitude) | Assessing the (expected) results of following the guideline. |
|  | 1. Emotion (Emotion) | Personal feelings influencing recommendation adherence. |
|  | 1. Reinforcement | Indicating expected consequences that impact their tendency for recommendation adherence. |
|  | 1. Intentions | Level of commitment to execution of the recommendation. |
|  | 1. Goals | Imagined outcomes that a cardiologist wants to achieve by following the recommendation. |
|  | 1. Optimism | Feeling confident that the recommendation will yield positive results. |
