# Supplementary material for: Barriers and facilitators to the uptake of the Ischaemia with Non-Obstructive Coronary Arteries (INOCA) recommendation by cardiologists in the Netherlands: A qualitative study
Source: Int J Cardiol Cardiovasc Risk Prev. 2025 Jul 29;27:200480. doi: 10.1016/j.ijcrp.2025.200480 (PMC12344192; doi:10.1016/j.ijcrp.2025.200480)
Supplement: Multimedia component 3 [file mmc3.docx]

**Supplement 3: Facilitators and barriers impacting recommendation uptake**

| **COM-B components** | **TDF-domain^[[1]](#footnote-2)^** | **Facilitators impacting recommendation uptake** | **Barriers impacting recommendation uptake** |
| --- | --- | --- | --- |
| **Capabilities (physical and psychological)** | 1. Knowledge [10] | - 1. The recommendation provides necessary (scientific) knowledge for a clear diagnostic and treatment process | - 1. Lack of familiarity with the (existence of the) recommendation   2. Existing (scientific) knowledge for diagnosis and treatment is insufficient in the recommendation |
|  | 1. Social/professional role and identity (Self-standards) [10] | - 1. Taking INOCA patients seriously is part of a cardiologists' professional role | - 1. A recommendation carries less weight than a guideline   2. Making the decision to diverge from the recommendation based on clinical judgement is part of a cardiologists' professional role   3. Involvement in INOCA care is (partly) guided by professional preferences |
|  | 1. Memory, attention and decision processes [9] | - 1. Tendency to revisit recommendation to remember relevant information   2. Concise information facilitates efficient use of recommendation   3. Revisiting recommendation is perceived as unnecessary for remembering relevant information | - 1. A mount of information impairs efficient use of recommendation |
|  | 1. Skills [8] | - 1. Implementation of the recommendation does not require new technical skills | - 1. Working with INOCA patients requires strong interpersonal skills |
|  | 1. Beliefs about capabilities (Self-efficacy) [7] | - 1. A cardiologists' confidence in their ability to communicate effectively with INOCA patients | - 1. Uncertainty about making clinically sound decisions regarding INOCA diagnosis and treatment   2. Having the ability to detect symptoms of INOCA in a patient's complex symptom presentation   3. Lacking interpersonal communication skills for effective communication with INOCA patients |
|  | 1. Behavioral regulation [5] | - 1. Checking recommendation in hindsight   2. Keeping the recommendation easily accessible   3. Team division of responsibility regarding INOCA care   4. Proactively scheduling INOCA patients at moments with more time |  |
| **Opportunities (physical and social)** | 1. Social influences (Norms) [11] | - 1. Team division/colleague with expertise on INOCA   2. Colleague in a superior hierarchical position determines the course on INOCA   3. Interaction with knowledgeable experts impacts recommendation uptake | - 1. Perceived complexity and characteristics of the patient group   2. The dominant image of an INOCA patient is a stereotyped woman and affects cardiologist perception and assessment of the patient population.   3. The predominance of men cardiologists impacts attention to a condition perceived as a women’s issue   4. Symptom presentation, risk factors and language are more focused on women with a potentially stigmatizing effect on men   5. The absence of attention to a (woman) patient’s context reduces optimal diagnosis and treatment   6. INOCA awareness created by women cardiologists resulted in resistance among some cardiologists |
|  | 1. Environmental context and resources (Environmental constraints) [9] |  | - 1. Patients with INOCA require additional time and attention, but the high throughput at the outpatient clinic places a significant demand on cardiologists' energy resources   2. Waiting times for tests or scans slow down the diagnostic and treatment process   3. There is a lack of multidisciplinary professionals that could support INOCA patients   4. The ICT facilities are insufficient in supporting efficient and effective consultations   5. Some suggested medications in the recommendations are not available in The Netherlands |
| **Motivation (reflective and automatic)** | 1. Beliefs about consequences (Anticipated outcomes/attitude) [10] | - 1. Improved clarity of diagnostic and treatment process for patients and cardiologists   2. Increased acknowledgement of INOCA (patients) among cardiologists | - 1. Insufficient follow-up care after INOCA diagnosis   2. Risk of unnecessary use of invasive procedures   3. Potential overdiagnosis of INOCA |
|  | 1. Emotion (Emotion) [5] |  | - 1. Consultations with (some) INOCA patients are emotionally draining   2. Delivering insufficient care for INOCA patients is frustrating |
|  | 1. Goals [3] | - 1. Helping the patient to accept the reality and impact of their diagnosis   2. Intrinsic curiosity and drive to clarify knowledge gaps and improve patient care |  |
|  | 1. Reinforcement [1] | - 1. Recommendation uptake leads to concrete patient improvement   2. Clinical practice stimulates being up to date about the INOCA recommendation |  |
|  | 1. Intentions [0] |  |  |
|  | 1. Optimism [0] |  |  |

1. The number behind the domain refers to the number of interviews where the domain was mentioned as relevant for uptake. [↑](#footnote-ref-2)
